# Effects of low-dose medium-chain triglycerides on bowel habit outcomes in Japanese adults prone to constipation: a randomized, double-blind, LCT-controlled crossover trial

**DOI:** 10.3389/fnut.2026.1746245

**Published:** 2026-03-11

**Authors:** Yuki Otsubo, Haruna Ishikawa, Keiichi Kojima, Shinji Watanabe, Naohisa Nosaka, Tsuneo Matsuike

**Affiliations:** 1 Strategic Invention R&D, The Nisshin OilliO Group, Ltd., Yokohama, Japan; 2 Matsuike Clinic, Tachikawa, Japan

**Keywords:** abdominal condition, bowel movements, constipation, decanoic acid, health-related quality of life, medium-chain fatty acids (MCFAs), medium-chain triglycerides (MCTs), octanoic acid

## Abstract

**Background:**

In Japan, many individuals experience symptoms of constipation. Medium-chain triglycerides (MCTs), composed solely of medium-chain fatty acids, have been suggested to improve bowel movements in athletes. However, most prior studies have assumed high-dose intake for ergogenic purposes, and evidence on the effects of low-dose interventions in generally healthy Japanese adults with a tendency toward constipation remains limited. In this study, we investigated the effects of consuming a small amount of MCTs on bowel movements, subjective abdominal condition, and health-related quality of life (HRQOL) in Japanese adults prone to constipation.

**Methods:**

This randomized, double-blind, long-chain triglyceride (LCT)-controlled, crossover trial with a 2-week washout enrolled 88 healthy Japanese adults aged 20–64 years with three to five bowel movements per week. Participants ingested 2 g/day of MCTs and 2 g/day of LCTs for 2 weeks each. Bowel movement indicators, subjective abdominal condition, and HRQOL were measured.

**Results:**

Using a statistical model for within-participant comparisons, significant diet effects were observed for the number of days with bowel movements, bowel movement frequency, and stool volume. A *post-hoc* analysis further confirmed that the change from baseline in the number of days with bowel movements at week 2 was significantly greater during the MCT intake period than during the LCT intake period. By contrast, although multiple questionnaire-based endpoints showed significant pre–post changes within intervention periods, the corresponding between-diet differences were not clearly demonstrated.

**Conclusion:**

Daily intake of even a small amount of MCTs, compared with LCTs, may be helpful in improving bowel movements in Japanese adults prone to constipation.

**Clinical trial registration:**

https://center6.umin.ac.jp/cgi-open-bin/ctr/ctr_view.cgi?recptno=R000060455; identifier UMIN000052997.

## 1 Introduction

Constipation is defined as “a condition characterized by the retention of feces in the large intestine, leading to pellet-like or hard stools, reduced bowel movement frequency, excessive straining, a sense of incomplete evacuation, anorectal blockage, or difficulty in defecation” (1). In Japan, approximately 36% of individuals reported some of the symptoms linked to constipation (2). Chronic constipation, which is defined as “a condition in which persistent constipation disrupts daily life and may lead to various physical impairments,” has been shown to be associated with reduced survival rates (3) and an increased risk of ischemic stroke and coronary artery disease (4). Additionally, chronic constipation negatively affects quality of life (QOL) (5, 6) and work productivity (7). Therefore, improving bowel movements in individuals prone to constipation as a preventive intervention is clinically significant, not only for promoting health but also for enhancing QOL and productivity.

Medium-chain fatty acids (MCFAs) are saturated fatty acids with carbon chain lengths ranging from 6 to 12. MCFAs form medium-chain triglycerides (MCTs), which are preferentially metabolized as energy substrates compared to long-chain triglycerides (LCTs), which are composed of long-chain fatty acids (LCFAs) (8). By leveraging this property, MCTs have been widely used as a nutritional supplement for patients with malabsorption and for preterm infants, among others (9). Recent evidence has suggested that MCTs may also contribute to improved bowel movements in athletes. For example, it was reported that rugby players who consumed approximately 18 g of MCTs daily for 2 months exhibited significant improvements in bowel movements, as assessed via subjective condition questionnaires (10). However, it remains unclear whether MCTs have a similar effect in populations with a tendency toward constipation. Furthermore, it is unknown whether the continuous intake of small amounts of MCTs, instead of the relatively large doses used in previous ergogenic studies, can improve bowel movements.

While changes in bowel habits as a side effect of consuming relatively large amounts of MCTs for nutritional purposes may negatively affect QOL, increased bowel frequency and stool volume in individuals prone to constipation could alleviate symptoms and improve their QOL. This study aimed to investigate whether the continuous intake of small amounts of MCTs (2 g/day) might improve bowel movements in a Japanese population with a tendency toward constipation, defined as having three to five bowel movements per week. Additionally, we examined the effects of MCT intake on subjective abdominal and health-related quality of life (HRQOL).

## 2 Materials and methods

### 2.1 Ethics statement

The trial was conducted in Tokyo, Japan, between February 27 and April 24, 2024. It was conducted under the supervision of a physician and a contract research organization (ORTHOMEDICO Inc., Tokyo, Japan) in accordance with the Declaration of Helsinki, the Ethical Guidelines for Medical and Biological Research Involving Human Subjects (11). The study was reviewed and approved by the ethical committee of the Takara Clinic, Medical Corporation Seishinkai (approval number: 2311–01400-0024-12-TC), and the details were registered at UMIN-CTR before the study commenced (UMIN-ID: UMIN000052997, URL: https://center6.umin.ac.jp/cgi-open-bin/ctr/ctr_view.cgi?recptno=R000060455).

### 2.2 Sample size

The sample size was set at 88 participants. As no previous interventional studies have investigated the effects of small amounts of MCTs on bowel movements in a Japanese population prone to constipation, the sample size was calculated with reference to a crossover study conducted in healthy Japanese adults, which reported a small effect size for the primary outcome, namely the number of days with bowel movements (12). From the mean difference and standard deviation of the number of days with bowel movements after 2 weeks of consuming the test and control foods, the required sample size was estimated to be at least 78 participants. To account for the anticipated dropouts and protocol deviations during the study period, the final sample size was adjusted to 88 participants.

### 2.3 Subjects

The participants were recruited from an enrollment bank. Screening included a medical interview, measurements of vital signs (systolic and diastolic blood pressure), height, weight, body mass index (BMI), urinalysis, peripheral blood tests, and the recording of bowel movement patterns in a diary during a 2-week screening period. Eligible participants were those who met the inclusion criteria, did not meet any of the exclusion criteria, and agreed to comply with the trial requirements during the study period.

The inclusion criteria were (1) Japanese males and females; (2) healthy individuals; (3) individuals aged between 20 and 64 years at the time of consent; (4) individuals with a bowel movement frequency of 3–5 times per week during the screening period; (5) individuals with regular meal times, consuming three meals per day; and (6) individuals with a BMI of less than 30 kg/m^2^.

Exclusion criteria were as follows: (1) individuals undergoing treatment for, or with a history of, malignant tumors, heart failure, myocardial infarction, or those with implanted pacemakers or implantable cardioverter defibrillators; (2) individuals undergoing treatment for arrhythmia, liver dysfunction, chronic kidney disease, cerebrovascular disease, rheumatic diseases, diabetes, dyslipidemia, hypertension, or other chronic diseases; (3) individuals deemed unsuitable for the study based on clinical test results or measurements during the screening period; (4) individuals who are pregnant, lactating, or planning to become pregnant during the study period; (5) individuals who smoke or quit smoking within 1 year prior to providing consent; (6) individuals who habitually consume excessive amounts of alcohol (≥60 g/day); (7) individuals who regularly consume foods for specified health uses (FOSHU), functional foods, medications (including herbal medicines), or supplements that may affect the study, and who cannot refrain from their use during the study period; (8) individuals who have consumed healthy oils (e.g., coconut oil, MCT oil) that may enhance the evaluated dietary components within the past 3 months; (9) individuals with known allergies to the ingredients of the test food or other substances; (10) individuals with diseases that significantly affect bowel movements or those who consume foods or ingredients that may influence bowel movements (e.g., yogurt, lactic acid beverages, foods fortified with dietary fiber, fermented foods, etc.) at least 4 days per week; (11) individuals with a body weight fluctuation of ±5 kg or more within the past 2 months, or those with extreme dietary habits; (12) individuals with irregular lifestyle rhythms due to night shifts, shift work, or extremely irregular eating or sleeping habits; (13) individuals whose work or other circumstances limit their opportunity to use the toilet when needed; (14) individuals who perform physical exercise aimed at maintaining or improving fitness at least twice a week for 30 min per session; (15) individuals, or their cohabiting family members, who are planning major life events (such as relocation, school enrollment, examinations, travel, or business trips) during the study period; (16) individuals living with an infant under 1 year of age, living with a person requiring nursing care, or sharing a bed with others due to caregiving or childcare responsibilities; (17) individuals who participated in other clinical studies within 28 days before providing consent, or those planning to participate in other studies during the trial period; (18) individuals judged as unsuitable for the study by the principal investigator.

### 2.4 Study design and allocation of participants

This study was conducted as a randomized, double-blind, LCT-controlled, crossover trial. A third-party organization not involved in the trial (allocation manager) randomly assigned participants to two groups: the control supplement-first group (Sequence 1) and the test supplement-first group (Sequence 2). The allocation table was generated using the R software, employing a stratified block randomization algorithm. The stratification factors were sex (male or female) and age (≥49 or <49 years) at the time of screening. The allocation manager securely maintained the allocation table until the key opening, and blinding was strictly maintained for all personnel involved in the trial except the allocation manager.

**Figure 1:**
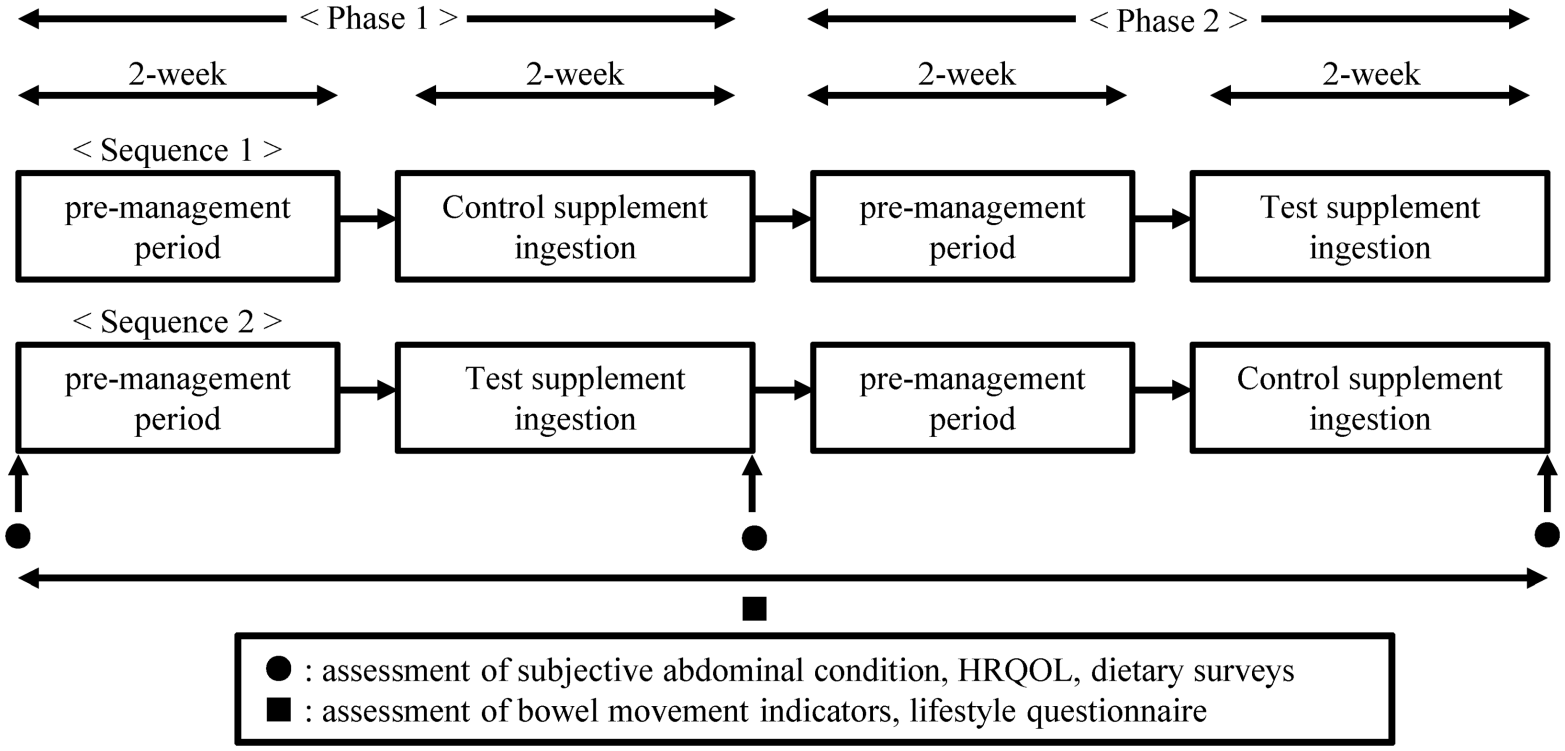
Outline of the study design.

The outline of the study design is shown in Figure 1. As no prior interventional studies have examined the effects of small amounts of MCTs on bowel movements in individuals with a tendency toward constipation, the study design was based on previous research conducted in healthy Japanese individuals prone to constipation (13, 14). Specifically, we implemented a 2-week supplement ingestion period, followed by a 2-week washout period. This washout duration was selected to minimize potential carryover effects and is consistent with methodological precedent. An evidence-based constipation guideline considered crossover studies eligible when the washout period was ≥2 weeks (or, if <2 weeks, only when first-period data could be extracted to reduce carryover risk) (15). Although our participants had a mild tendency toward constipation rather than chronic constipation, we conservatively adopted this ≥2-week washout benchmark to mitigate carryover in bowel-habit endpoints. In addition, regulatory trial design guidance for spontaneous bowel movements (SBMs)-based constipation/IBS-C endpoints explicitly includes a 2-week baseline/washout period prior to treatment allocation (16). Mechanistically, MCTs are predominantly transported to the liver via the portal vein and are rapidly *β*-oxidized, making prolonged residual effects biologically unlikely (17); consistent with this, randomized crossover studies of MCT ingestion for non-bowel outcomes have used 2-week intervention periods separated by 2-week washouts (18). After a 2-week pre-management period (defined as the phase 1 pre-management period), participants consumed the first supplement for 2 weeks (defined as the phase 1 supplement intake period). This was followed by a 2-week washout (defined as the phase 2 pre-management period). Thereafter, participants consumed another supplement for 2 weeks (defined as the phase 2 supplement intake period). The intervention period was defined as the time from the start date of the phase 1 pre-management period to the end date of the phase 2 supplement intake period. During the supplement intake period, participants consumed one sachet of the supplement (10 g) once daily after breakfast. If the sachet was not consumed after breakfast, participants were instructed to consume it after lunch and record this in their diary. If it was not consumed after lunch, they were instructed to consume it after dinner and record this in their diary. If a sachet was missed for the day, it was not to be taken the following day (no carryover), and the missed dose was to be recorded in the diary. Throughout the study, participants were instructed to adhere to compliance requirements, as described in the next section.

### 2.5 Compliance requirements

(1) Participants must consume the supplement as instructed, following the specified method and dosage, and ensure a compliance rate of at least 80%. (2) Participants must avoid consuming foods, beverages, or supplements categorized as FOSHU, Foods with Function Claims, or other functional foods that may affect the study. (3) Participants with a pre-existing habit of consuming unrestricted health foods or supplements must record their intake in the provided diary and maintain their usual intake habits. Participants without such habits must not start consuming new health foods or supplements during the study period. (4) Participants may continue consuming foods they habitually consume, even if these foods contain ingredients that may influence bowel movements or the study’s target area (e.g., yogurt, lactic acid beverages, foods fortified with dietary fiber or oligosaccharides, milk, butter, ice cream, cheese, high-fat dairy products, other fermented foods, health foods, or supplements). However, participants must not significantly change their consumption quantity or frequency. Any changes must be recorded in the provided diary. (5) Participants must avoid consuming foods that may enhance the evaluated dietary components (e.g., coconut oil, MCT oil). (6) From the date of consent to the completion of the phase 2 supplement intake period, participants must maintain their usual lifestyle and avoid excessive exercise, extreme dieting, overeating, or excessive alcohol consumption. (7) From the day before the screening visit until the end of the measurements, and from the day before the questionnaire evaluation until its completion, participants must avoid alcohol consumption and strenuous exercise. (8) Participants must immediately report any changes in physical condition during the study period to the contract research organization and follow its instructions for subsequent actions.

### 2.6 Supplement

The supplement was provided in the form of stick-pack jelly. LCTs (rapeseed oil from The Nisshin OilliO Group, Ltd., Tokyo, Japan) were used in the control supplement, while MCTs (Nisshin MCT oil from The Nisshin OilliO Group, Ltd., Tokyo, Japan) were used in the test supplement. Each stick-pack jelly weighed 10 g. Minimal differences occurred in the composition or content of gelling agents, emulsifiers, seasonings, or flavorings between the control and test supplement. Table 1 shows the minimum and maximum amounts of lipids as well as their functional components, i.e., octanoic acid (C8) and decanoic acid (C10), associated with the daily intake of supplements.

**Table 1:** Contents of LCTs, MCTs, and MCFAs in 10 g of supplement.

Components	Unit	Control supplement	Test supplement
LCTs	g/day	2	-
MCTs	g/day	-	2
Octanoic acid (8:0)	g/day^1^	-	(1.24, 1.38)
Decanoic acid (10:0)	g/day^1^	-	(0.352, 0.487)

1 Lower and upper limits are shown.

-, Content is zero.

### 2.7 Lifestyle survey

During the intervention period, participants were instructed to record their daily lifestyle information in a lifestyle survey form. This included body weight, presence or absence of exercise, alcohol consumption, number and quantity of meals, sleep duration, changes in physical condition, and supplement intake.

### 2.8 Adverse events

Adverse events were defined as the occurrence of “new abnormalities” or the “worsening of existing conditions” that were clinically significant in terms of physical signs or symptoms during the study period. If an adverse event was observed, the principal investigator assessed the causal relationship between the event and the supplement.

### 2.9 Dietary survey

Energy and nutrient intake were estimated using a brief-type self-administered diet history questionnaire (BDHQ). The questionnaire was provided on the first day of the phase 1 pre-management period, on the end of the phase 1 supplement intake period, and on the end of the phase 2 supplement intake period.

### 2.10 Outcomes

The primary outcomes were defined as the number of days with bowel movements and the bowel movement frequency. The secondary outcomes included stool volume, stool consistency, stool color, stool odor, the feeling of complete evacuation, the subjective abdominal condition, and HRQOL.

### 2.11 Assessment of bowel movement indicators

Bowel movement indicators were assessed using a web-based diary. The evaluation items included the number of days with bowel movements, bowel movement frequency, stool volume, stool consistency, stool color, stool odor, and the feeling of complete evacuation. Participants were required to record their defecation status in the diary daily during the 2-week screening period and the intervention period. Participants were instructed to record their daily results at the end of each day. The number of days with bowel movements (days per week) and the bowel movement frequency (times per week) were evaluated based on the recorded entries in the diary. The stool volume (containers per week) was assessed using the size of the film-type containers No.7 (Roppon-Ashi Entomological Books, Tokyo, Japan). Participants estimated the equivalent number of containers that corresponded to their stool volume. The stool consistency was evaluated using the Bristol Stool Scale (19). The stool color was assessed using the following numerical scale: 1; yellow, 2; ochre, 3; brown, 4; dark brown, 5; chestnut brown, 6; black brown. The stool odor was evaluated using the following numerical scale: 1; very strong odor, 2; slightly strong odor, 3; no change from usual, 4; slightly weaker odor, 5; very weak odor. The feeling of complete evacuation was assessed using the following numerical scale: 1; feeling of relief, 2; slightly relieved feeling, 3; neutral feeling (neither relieved nor dissatisfied), 4; slight feeling of incomplete evacuation, 5; strong feeling of incomplete evacuation. For the number of days with bowel movements, the bowel movement frequency, and the stool volume, the actual values were calculated as the weekly total during the supplement intake period and defined as week 1 and week 2 data. For stool consistency, stool color, stool odor, and the feeling of complete evacuation, the actual values were calculated as the weekly average during the supplement intake period, rounded to the nearest integer (based on the first decimal place), and defined as week 1 and week 2 data. For each of these outcomes, the change value was defined as the weekly value during the supplement intake period minus the weekly value during the second week of the immediately preceding pre-management period (defined as week 0). Week 0 was used as the baseline because the first week of the phase 2 pre-management period included data from the day following the phase 1 supplement intake period, potentially carrying over residual effects.

### 2.12 Assessment of subjective abdominal condition

The degree of subjective abdominal condition was evaluated using a Visual Analog Scale (VAS) for parameters including the feeling of incomplete evacuation, abdominal condition, lower abdominal bloating, stress, and irritability. A 10 cm horizontal line was used, with the left end defined as “the best condition ever experienced” and the right end defined as “the worst condition ever experienced.” Participants were asked to mark a vertical line on the horizontal scale to indicate their current abdominal condition at the time of assessment. The assessment was conducted on the first day of the phase 1 pre-management period, at the end of the phase 1 supplement intake period, and at the end of the phase 2 supplement intake period. For the abdominal condition data, actual values were obtained at baseline (the first day of the phase 1 pre-management period), at the end of the phase 1 supplement intake period, and at the end of the phase 2 supplement intake period. Change values were calculated as the difference from baseline at each post-baseline time point.

### 2.13 Assessment of HRQOL

Subjective health perception was assessed using the SF-36v2 (Standard Version), a health-related quality of life (HRQOL) questionnaire. The assessment was conducted on the first day of the phase 1 pre-management period, at the end of the phase 1 supplement intake period, and at the end of the phase 2 supplement intake period. For the HRQOL data, actual values were obtained at baseline (the first day of the phase 1 pre-management period), at the end of the phase 1 supplement intake period, and at the end of the phase 2 supplement intake period. Change values were calculated as the difference from baseline at each post-baseline time point.

### 2.14 Statistical analysis

Statistical analyses were performed using the following methods.

The nutrient intake was assessed for normality using the Shapiro–Wilk test. If normality was not satisfied, the Mann–Whitney U test was applied. If normality was satisfied, homoscedasticity was assessed using the F-test. If homoscedasticity was confirmed, the Student’s t-test was used; otherwise, the Welch’s t-test was applied.

Statistical analyses for the Bowel Movement Indicators, Subjective Abdominal Condition, and HRQOL data were performed as follows. First, a linear mixed-effects model with diet, phase, and sequence as fixed effects and subjects as a random effect was used to examine the validity of the crossover design and the presence of a dietary effect. For outcomes with baseline data in each phase (bowel movement indicators) in the model for actual values, the baseline data for each phase were additionally included as a covariate. For bowel movement indicators with time-course data (week 1 and week 2), a linear mixed-effects model with diet and time (week) as fixed effects and subjects as random effects was used to examine the interaction between diet and time.

For outcomes in which phase and sequence effects were not observed and dietary effects were observed, between-diet differences between the test supplement and control supplement were evaluated using both actual values and change values. Normality was assessed using the Shapiro–Wilk test. If normality was not satisfied, the Mann–Whitney U test was applied. If normality was satisfied, homoscedasticity was assessed using the F-test. If homoscedasticity was confirmed, the Student’s t-test was used; otherwise, the Welch’s t-test was applied.

As a supplementary analysis, within-group comparison between the baseline and post-intervention values was performed. For outcomes with time-course data, normality and homoscedasticity were assessed; if neither was significant, Dunnett’s test was conducted, whereas if either was significant, Steel’s test was used. For outcomes without time-course data, normality and homoscedasticity were assessed. If normality was not satisfied, the Mann–Whitney U test was applied. If normality was satisfied, homoscedasticity was assessed using the F-test. If homoscedasticity was confirmed, the Student’s t-test was used; otherwise, the Welch’s t-test was applied.

Descriptive statistics for the analyzed data were calculated using Microsoft Excel for Office 365 MSO (Microsoft Japan Co., Ltd., Tokyo, Japan), while statistical analyses were performed using the R statistical software, version 4.1.0 for Windows (R Core Team, Vienna, Austria). A significance level of less than 5% was considered statistically significant.

## 3 Results

### 3.1 Analysis of subjects

**Figure 2:**
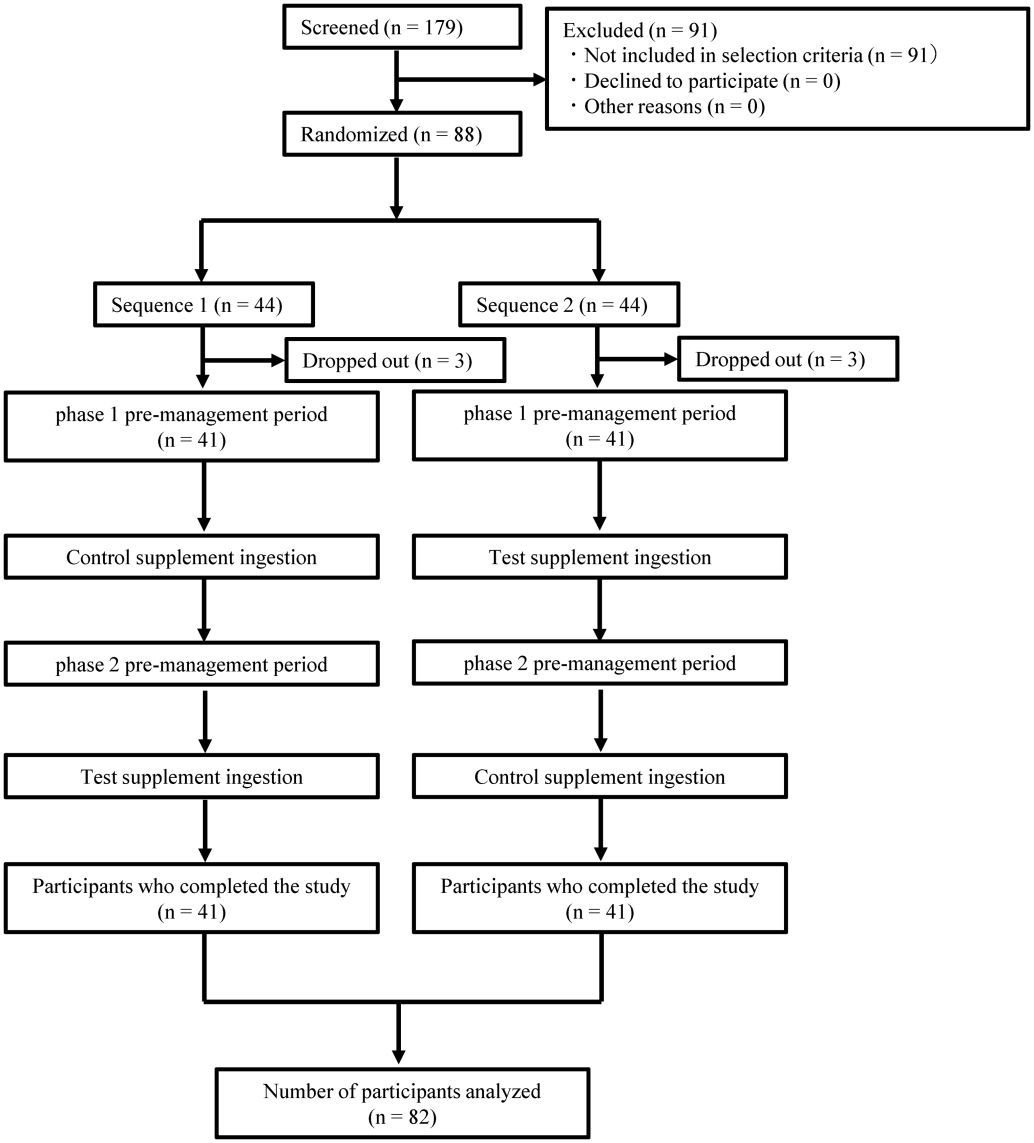
Flowchart of the study.

The eligibility was assessed for 179 individuals who provided informed consent, and 88 individuals were enrolled in the study. These participants were randomly assigned to two groups, specifically, 44 to the control supplement-first group (Sequence 1) and 44 to the test supplement-first group (Sequence 2). Three participants in Sequence 1 and three participants in Sequence 2 did not receive the allocated intervention and had no efficacy data available. For participants with no valid data, submission of diaries had been delayed since the early phase of the intervention. By the time the contract research organization was able to establish contact, it was confirmed that they had not consumed the study food. Consequently, no data were available for these individuals. Thus, these six participants were excluded from the analysis, leaving a total of 82 participants (Figure 2). The baseline characteristics of the analyzed participants are summarized in Table 2.

**Table 2:** Baseline clinical characteristics of the participants (n = 82).

Characteristics	Unit	Values
Number of subjects (M/F)		82 (37/45)
Age	Years	47.3 ± 10.6
Height	cm	164.2 ± 7.0
Body weight	kg	61.4 ± 11.1
BMI	kg/m^2^	22.6 ± 3.1
Number of days with bowel movements	days/week	3.73 ± 0.93
Bowel movement frequency	times/week	3.91 ± 0.96
Stool volume	containers/week	18.28 ± 16.06
Stool consistency	-	3.34 ± 1.16
Stool color	-	3.58 ± 0.82
Stool odor	-	2.88 ± 0.36
Feeling of complete evacuation	-	2.37 ± 1.25

Values are shown as mean ± standard deviation. BMI, body mass index. The values for the bowel movement indices are the mean values over the two-week screening period.

### 3.2 Adverse events

No adverse events attributable to the ingestion of the supplement were observed during the intervention period.

### 3.3 Nutrient intake

The energy and nutrient intake (excluding the supplements) during the study period is shown in Table 3. No significant differences were observed between the control supplement intake and the test supplement intake period.

**Table 3:** Mean daily nutrient intake (excluding the supplements; *n* = 82).

Nutrients	Unit	Control	Test
Energy	kcal/day	1370.6 ± 410.1	1356.8 ± 388.7
Water	g/day	1135.6 ± 446.6	1136.9 ± 424.0
Carbohydrate	g/day	187.7 ± 68.3	183.1 ± 59.8
Protein	g/day	50.0 ± 15.9	50.9 ± 15.9
Fat	g/day	41.3 ± 14.2	41.4 ± 13.9
Saturated fatty acid	g/day	10.2 ± 3.9	10.2 ± 3.6
Monounsaturated fatty acid	g/day	15.2 ± 5.4	15.2 ± 5.2
Polyunsaturated fatty acid	g/day	10.6 ± 3.7	10.8 ± 3.9
Octanoic acid (8:0)	mg/day	66.9 ± 54.4	61.2 ± 50.1
Decanoic acid (10:0)	mg/day	101.8 ± 80.3	92.8 ± 73.6
Fiber	g/day	8.5 ± 3.8	8.4 ± 3.6

Values are shown as mean ± standard deviation.

### 3.4 Bowel movement indicators

The results of the model-based analysis of bowel movement indicators, examining the diet effect, sequence effect, phase effect, and the time-by-diet interaction, are shown in Table 4. Significant diet effects were observed for number of days with bowel movements, bowel movement frequency, and stool volume. The results were consistent in the model using change values. No sequence effect was observed for any indicator. A phase effect was observed for stool color and stool odor. No time-by-diet interaction was observed for any indicator. *Post-hoc* between-diet comparisons for indicators with a significant diet effect are presented in Table 5. The change value of number of days with bowel movements in week 2 was significantly higher with MCT intake compared with LCT intake. In addition, as a supplemental pre- vs. post-intervention comparison, both the test supplement and the control supplement significantly increased number of days with bowel movements, bowel movement frequency, and stool volume, and significantly softened stool consistency. Although the mean stool volume at week 2 during the control intervention was smaller than at week 0, individual-level data showed that 45 participants experienced an increase, 10 showed no change, and 27 experienced a decrease relative to week 0, indicating that more participants had increased stool volume. Since the magnitude of decrease among those with reductions was relatively large, the mean change was negative. However, we employed nonparametric tests for within-group comparisons. Thus, the inferential results depended on the counts of individuals with increases vs. decreases rather than the magnitude of change itself.

**Table 4:** *p* values for the effects of diet, sequence, phase, and the diet × time interaction on bowel movement indicators (*n* = 82).

Outcomes	Model used actual value				Model used change value			
	Diet	Sequence	Phase	Interaction (diet × time)	Diet	Sequence	Phase	Interaction (diet × time)
Number of days with bowel movements (days/week)	0.0002	0.3782	0.2752	0.2158	0.0001	0.2130	0.2214	0.2602
Bowel movement frequency (times/week)	0.0006	0.3697	0.4087	0.6315	0.0008	0.1397	0.5452	0.6431
Stool volume (containers/week)	0.0019	0.2064	0.9901	0.4547	0.0018	0.0660	0.6171	0.5119
Stool consistency	0.4921	0.9192	0.4817	0.3271	0.3254	0.8810	0.4958	0.4030
Stool color	0.5402	0.8083	0.1692	0.4431	0.1420	0.5274	0.0364	0.5326
Stool odor	0.2334	0.7841	0.6687	0.4742	0.3674	0.1874	0.0481	0.5917
Feeling of complete evacuation	0.5176	0.5734	0.4571	0.8429	0.4778	1.0000	0.5944	0.8593

**Table 5:** Results of bowel movement indicators (*n* = 82).

Outcomes	Week	Actual value		Change value	
		Control	Test	Control	Test
Number of days with bowel movements (days/week)	Week 0	4.41 ± 0.19	4.24 ± 0.19		
	Week 1	4.67 ± 0.17	4.85 ± 0.19 †	0.26 ± 0.15 †	0.61 ± 0.14 †
	Week 2	4.38 ± 0.17	4.84 ± 0.19 †	−0.04 ± 0.15	0.60 ± 0.14 *†
Bowel movement frequency (times/week)	Week 0	4.84 ± 0.24	4.83 ± 0.28		
	Week 1	5.12 ± 0.23	5.61 ± 0.35	0.28 ± 0.19 †	0.78 ± 0.20 †
	Week 2	4.80 ± 0.19	5.45 ± 0.31	−0.04 ± 0.19	0.62 ± 0.21 †
Stool volume (containers/week)	Week 0	26.85 ± 3.82	25.08 ± 2.85		
	Week 1	27.83 ± 3.67	29.48 ± 3.72	0.98 ± 1.09 †	4.40 ± 1.53 †
	Week 2	26.00 ± 3.53	29.45 ± 4.07	−0.85 ± 1.94 †	4.38 ± 2.28 †
Stool consistency	Week 0	3.61 ± 0.11	3.67 ± 0.11		
	Week 1	3.94 ± 0.11	3.85 ± 0.11	0.33 ± 0.10 †	0.18 ± 0.08 †
	Week 2	3.73 ± 0.11	3.78 ± 0.09	0.12 ± 0.09	0.11 ± 0.10
Stool color	Week 0	3.38 ± 0.08	3.49 ± 0.08		
	Week 1	3.32 ± 0.08	3.38 ± 0.08	−0.06 ± 0.06	−0.11 ± 0.07
	Week 2	3.37 ± 0.08	3.35 ± 0.07	−0.01 ± 0.06	−0.13 ± 0.07
Stool odor	Week 0	2.90 ± 0.03	2.90 ± 0.04		
	Week 1	2.96 ± 0.03	2.91 ± 0.03	0.06 ± 0.04	0.01 ± 0.04
	Week 2	2.96 ± 0.03	2.95 ± 0.03	0.06 ± 0.04	0.05 ± 0.04
Feeling of complete evacuation	Week 0	3.07 ± 0.09	3.10 ± 0.09		
	Week 1	3.02 ± 0.09	2.99 ± 0.09	−0.05 ± 0.08	−0.11 ± 0.06
	Week 2	2.96 ± 0.09	2.95 ± 0.09	−0.11 ± 0.08	−0.15 ± 0.07

Values are given as mean ± standard error. *Significantly different from control supplement intake period (*p* < 0.05, Mann–Whitney U test). † Significantly different from the baseline (*p* < 0.05, Steel’s test).

### 3.5 Subjective abdominal condition

For Subjective Abdominal Condition, the results of the model examining the diet effect, sequence effect, and phase effect are shown in Table 6. No significant diet effect was observed for any indicator. A significant sequence effect was observed for the actual values of stress. In addition, a phase effect was observed for feeling of incomplete evacuation, stress, and irritability. The results of the supplemental pre- vs. post-intervention comparisons are shown in Table 7. For both actual and change values, all indices were significantly lower after intake than before intake in both the control and test supplements.

**Table 6:** *p* values for the effects of diet, sequence and phase on abdominal condition (*n* = 82).

Outcomes	Model used actual value			Model used change value		
	Diet	Sequence	Phase	Diet	Sequence	Phase
Feeling of incomplete evacuation	0.4290	0.1773	0.0462	0.4290	0.3528	0.0462
Abdominal condition	0.8586	0.0687	0.2502	0.8586	0.1681	0.2502
Lower abdominal bloating	0.9888	0.0773	0.5461	0.9888	0.1205	0.5461
Stress	0.6347	0.0203	0.0080	0.6347	0.2682	0.0080
Irritability	0.4447	0.0634	0.0317	0.4447	0.8270	0.0317

**Table 7:** Results of abdominal condition (*n* = 82).

Outcomes	Base	Actual value		Change value	
		Control	Test	Control	Test
Feeling of incomplete evacuation	53.6 ± 2.0	40.3 ± 1.7 †	38.9 ± 1.8 †	−13.3 ± 2.3 †	−14.7 ± 2.5 †
Abdominal condition	52.8 ± 1.9	39.4 ± 1.6 †	39.1 ± 1.8 †	−13.4 ± 2.2 †	−13.7 ± 2.0 †
Lower abdominal bloating	51.7 ± 2.4	36.5 ± 2.1 †	36.5 ± 2.0 †	−15.1 ± 2.0 †	−15.1 ± 2.4 †
Stress	52.6 ± 1.9	41.6 ± 2.1 †	40.9 ± 2.0 †	−11.0 ± 2.1 †	−11.8 ± 2.2 †
Irritability	47.5 ± 2.2	40.1 ± 2.3 †	38.7 ± 2.3 †	−7.3 ± 2.4 †	−8.8 ± 2.3 †

Values are given as mean ± standard error. † Significantly different from the baseline (*p* < 0.05, Mann–Whitney U test or Student’s t-test).

### 3.6 HRQOL

For HRQOL, the results of the model examining the diet effect, sequence effect, and phase effect are shown in Table 8. A significant diet effect was observed for Role-social component summary (RCS). In addition, a significant phase effect was observed for Social functioning (SF) and Mental component summary (MCS). In the model using actual values, significant sequence effects were observed for General health (GH), Vitality (VT), Mental health (MH), and Mental component summary (MCS). *Post-hoc* between-diet comparisons for Role-social component summary (RCS), which showed a significant diet effect, are presented in Table 9. No significant differences were observed in the between-diet comparisons. The results of the supplemental pre- vs. post-intervention comparisons are also shown in Table 9. In the case of the within-group comparison using change values, the intake of the control supplement significantly increased the scores for PF (Physical Functioning) and BP (Bodily Pain). Furthermore, the intake of the test supplement significantly increased the scores for RP (Role-Physical) and MH (Mental Health).

**Table 8:** *p* values for the effects of diet, sequence and phase on HRQOL (*n* = 82).

Outcomes	Model used actual value			Model used change value		
	Diet	Sequence	Phase	Diet	Sequence	Phase
Physical functioning (PF)	0.7068	0.6916	0.2030	0.7068	0.8748	0.2030
Role-physical (RP)	0.2123	0.4309	0.1701	0.2123	0.5595	0.1701
Bodily pain (BP)	0.0529	0.4936	0.4476	0.0529	0.0659	0.4476
General health (GH)	0.8012	0.0236	0.3346	0.8012	0.8000	0.3346
Vitality (VT)	0.3461	0.0131	0.5731	0.3461	0.5135	0.5731
Social functioning(SF)	0.6153	0.2358	0.0210	0.6153	0.8162	0.0210
Role-emotional (RE)	0.1775	0.4426	0.1775	0.1775	0.0522	0.1775
Mental health (MH)	0.3181	0.0231	0.1119	0.3181	0.5840	0.1119
Physical component summary (PCS)	0.0758	0.8945	0.8037	0.0758	0.5449	0.8037
Mental component summary (MCS)	0.9561	0.0042	0.0466	0.9561	0.3721	0.0466
Role-social component summary (RCS)	0.0347	0.9820	0.7311	0.0347	0.8130	0.7311

**Table 9:** Results of HRQOL (*n* = 82).

Outcomes	Base	Actual value		Change value	
		Control	Test	Control	Test
Physical functioning (PF)	90.9 ± 1.4	92.4 ± 1.3	92.1 ± 1.4	1.5 ± 0.9 †	1.2 ± 1.1
Role-physical (RP)	87.0 ± 1.9	88.5 ± 1.9	90.0 ± 1.7	1.5 ± 1.3	3.0 ± 1.3 †
Bodily pain (BP)	77.3 ± 2.3	81.5 ± 2.3	77.1 ± 2.5	4.2 ± 2.1 †	−0.2 ± 2.3
General health (GH)	64.2 ± 1.9	64.9 ± 1.9	65.2 ± 1.8	0.8 ± 1.1	1.0 ± 1.1
Vitality (VT)	57.0 ± 1.8	56.2 ± 2.1	57.3 ± 1.9	−0.8 ± 1.1	0.4 ± 1.2
Social functioning(SF)	85.7 ± 2.2	86.1 ± 2.2	87.0 ± 2.1	0.5 ± 2.2	1.4 ± 2.1
Role-emotional (RE)	85.6 ± 2.1	85.2 ± 2.1	86.9 ± 2.0	−0.4 ± 1.7	1.3 ± 1.7
Mental health (MH)	68.0 ± 1.6	68.8 ± 1.9	70.1 ± 1.7	0.9 ± 1.3	2.1 ± 1.3 †
Physical component summary (PCS)	52.2 ± 0.8	53.6 ± 0.8	52.5 ± 0.8	1.4 ± 0.7	0.3 ± 0.7
Mental component summary (MCS)	51.0 ± 0.8	51.2 ± 1.0	51.2 ± 0.9	0.1 ± 0.5	0.1 ± 0.6
Role-social component summary (RCS)	49.5 ± 0.9	49.2 ± 0.9	50.5 ± 0.8	−0.3 ± 0.8	1.0 ± 0.8

Values are given as mean ± standard error. † Significantly different from the baseline (*P* < 0.05, Mann–Whitney U test).

## 4 Discussion

According to the international guideline for functional gastrointestinal disorders known as Rome IV criteria, fewer than 3 spontaneous bowel movements per week is one of the key diagnostic criteria for chronic constipation (20). In this study, we defined individuals with 3–5 bowel movements per week as having a “tendency toward constipation” and evaluated the effect of MCT intake on bowel function in Japanese adults aged between 20 and 64 years who met this definition. Using a statistical model to compare dietary effects within the same participants, significant diet effects were observed for three endpoints: number of days with bowel movements, bowel movement frequency, and stool volume. In addition, a *post-hoc* analysis confirmed that the change from baseline in the number of days with bowel movements at week 2 was significantly greater during the MCT intake period than during the LCT intake period. Collectively, these findings suggest that even a small amount of MCT (2 g/day) may contribute to improving bowel habits in constipation-prone Japanese adults. Importantly, this trial was not a therapeutic intervention in patients with chronic constipation but rather an evaluation of bowel habit improvement in constipation-prone individuals. In such a population, because baseline bowel frequency is not extremely low, ceiling effects and substantial intra-individual variability may attenuate group-level effects, and between-diet differences in mean changes may appear modest. Consistent with this, compared with the control diet, the test diet was associated with modest mean increases of approximately 0.64 days/week in bowel movement days and 0.66 times/week in bowel movement frequency. However, this magnitude of improvement is in line with the order of effect sizes reported in original studies of food ingredients and pro−/post-biotics aimed at improving bowel habits. For example, in a placebo-controlled crossover study of maltobionic acid (4 g/day for 4 weeks), changes in bowel movement days were +1.33 days/week with the test food and +0.67 days/week with placebo, corresponding to an incremental effect of approximately +0.66 days/week (21). In addition, in a placebo-controlled study of heat-killed *Bifidobacterium longum* CLA8013 (2 weeks) in constipation-prone healthy adults (3–5 bowel movements/week), changes in bowel movement frequency were +1.16 times/week with the intervention and +0.51 times/week with the control, corresponding to an incremental effect of approximately +0.65 times/week (22). Taken together, these data indicate that the between-diet differences observed in the present study can be situated within the range reported for food-based interventions in healthy, constipation-prone populations.

Prior studies have reported the beneficial effects of MCTs on bowel function. Among corporate rugby players who consumed 18 g/day of MCTs for 2 months, a questionnaire on subjective condition showed a significant improvement in bowel habits compared with a control group without MCT intake (10). Similarly, among female collegiate soccer players who consumed 18 g/day of MCTs for 6 weeks, the number of weekly bowel movements increased significantly compared with pre-intake values (23). Despite differences in dose, duration, and study population, these findings align with the present results.

Because mechanistic biomarkers were not assessed in the present study, the mechanisms underlying the observed improvement in bowel habits cannot be identified; however, several plausible pathways may be considered. First, in the previously conducted study on female collegiate soccer players, an increased production of short-chain fatty acids (SCFAs) associated with alterations in the gut microbiota was proposed as a potential mechanism because SCFAs may improve bowel habits by enhancing intestinal motility (23). However, conflicting findings have also been reported regarding microbiota responses to MCT intake (10), suggesting that these effects may be condition-dependent. Second, MCT intake has been reported to increase circulating acylated ghrelin concentrations (24–27), which may promote bowel movements by altering gastric motility and gastric emptying and thereby modulating the gastrocolic reflex. Nevertheless, because the MCT dose administered in the present study was lower than those used in previous studies, the contribution of this pathway should be regarded as speculative. Third, bile acids can stimulate colonic water and electrolyte secretion and colonic motility, thereby potentially affecting bowel movement frequency and ease of defecation (28–31). Some animal studies have further suggested that medium-chain fatty acids may increase bile acid synthesis and secretion and reduce ileal reabsorption, resulting in increased fecal bile acid excretion (32, 33), which could increase bile acid delivery to the colon and contribute to improved bowel habits. Taken together, the improvement in bowel habits associated with MCT intake may involve multiple pathways; however, these mechanisms remain hypothetical at present. Future studies incorporating biomarkers such as the gut microbiota, SCFAs, acylated ghrelin, and bile acids are warranted.

In this study, we evaluated the abdominal condition and subjective health perception, using a VAS questionnaire and the SF-36. The SF-36 is among the most widely used instruments for assessing subjective health status worldwide, and its reliability and validity have been scientifically verified in Japan. The SF-36 measures subjective health across eight subscales: Physical Functioning (PF), Role-Physical (RP), Bodily Pain (BP), General Health (GH), Vitality (VT), Social Functioning (SF), Role-Emotional (RE), and Mental Health (MH). It is also common to summarize these into three component scores: the Physical Component Summary (PCS; composite of PF, BP, GH, RP, SF, VT), the Mental Component Summary (MCS; composite of BP, GH, SF, RE, VT, MH), and the Role/Social Component Summary (RCS; composite of BP, GH, RP, SF, RE) (34, 35). Using a within-subject model for between-diet comparisons, a significant diet effect was observed only for the SF-36 Role/Social Component Summary (RCS), whereas no clear between-diet differences were demonstrated for the other outcomes. In addition, when examining pre–post changes during the MCT intervention period, restricted to measures for which no sequence effect or phase effect was suggested, significant improvements were observed in the VAS items abdominal condition and lower abdominal bloating, as well as in the SF-36 subscales Role-Physical (RP) and Mental Health (MH). Patients with chronic constipation are known to have lower quality of life (QOL) than individuals without constipation (5, 6), and alleviating constipation has been reported to improve QOL (36). Given that bowel movements improved to some extent with continuous MCT intake in the present study, it is plausible that improvements in bowel habits and bowel-related subjective symptoms contributed to changes in these QOL-related measures. However, although the RCS showed a significant diet effect in the model, *post-hoc* analyses did not confirm a significant between-diet difference. Moreover, because sequence effects and/or phase effects were observed for multiple endpoints, these QOL-related findings should be interpreted cautiously and are best regarded as exploratory, avoiding overinterpretation.

In this trial, MCT intake was associated with an increase in the number of bowel movement days relative to the LCT intake. Meanwhile, focusing on within-group changes during the control supplement intake period, the LCT intervention led to significant increases in defecation indices, namely, number of defecation days, defecation frequency, and stool volume, along with a significant softening of stool consistency. With respect to questionnaire-based outcomes, among the items for which pre–post changes were observed, and restricted to those for which no sequence effect or phase effect was detected, significant improvements were observed in the VAS items abdominal condition and lower abdominal bloating. Similarly, for the SF-36, significant improvements were observed in the physical-domain subscales Physical Functioning (PF) and Bodily Pain (BP). Similar to the MCT condition, these improvements in VAS and SF-36 scores may have been driven by LCT-induced improvements in bowel function. The observation of improvements under the control condition suggests that the control may not have been physiologically inert. Long-chain triglycerides can strongly stimulate gallbladder contraction and cholecystokinin (CCK) secretion via long-chain fatty acids, and bile acids can influence stool characteristics and bowel movement frequency by promoting colonic secretion and motility. Therefore, the potential contribution of physiological effects associated with a fat-based control condition should be considered. Regarding LCT and bowel movements, one report in obese patients with chronic constipation found that delivering LCT to the distal small intestine using microcapsules improved constipation (37). The authors proposed that, akin to the laxative effect observed with the pancreatic lipase inhibitor, orlistat (38), facilitated transit of undigested fat into the colon may have contributed to constipation relief. However, in the present study, only a small amount of oil (2 g) was ingested orally, making the colonic delivery of undigested fat unlikely. Another report showed that, in constipated patients undergoing hemodialysis, the consumption of an average of either 5.7 mL/day of olive oil or 6.9 mL/day of flaxseed oil for 4 weeks improved bowel movements, similar to the use of mineral oil as a laxative (39). To the best of our knowledge, however, no prior study has reported that the continuous intake of as little as 2 g of LCTs improves bowel movements in individuals with a tendency toward constipation. Accordingly, given the dose used in our design, we considered it unlikely that LCTs could improve bowel movements and therefore selected it as the control. Nevertheless, our findings newly suggest that continuous intake of such a small amount (2 g) of LCTs may improve bowel movements in constipation-prone individuals. Importantly, the estimand in this study was not the effect vs. no intervention, but the incremental effect of MCT under the LCT condition. Accordingly, the observed between-diet differences in bowel outcomes and responder rates suggest an additional bowel-regulating effect of MCT in the context of the physiological effects of LCT. If the control condition itself exerted bowel habit–improving effects, the between-diet contrast would be expected to be attenuated; therefore, the estimates obtained in this trial may be conservative. Future studies should incorporate measures such as bile acids to investigate chain length–dependent mechanisms, and should employ a carbohydrate-based control or a fat-free control to more clearly evaluate the bowel habit–improving effects of dietary fat itself in individuals with a tendency toward constipation.

MCTs are used as nutritional supplements. However, they are known to sometimes cause symptoms such as diarrhea and loose stools (40–42). In the present study, no such adverse events that could be associated with the test supplement were observed. Therefore, with a small dose of 2 g of MCTs in individuals prone to constipation, the likelihood of adverse effects such as diarrhea or loose stools appears to be low. In addition, it has been reported that MCTs are more likely than LCTs to cause upper abdominal discomfort (43). In our trial, the mean SF-36 Bodily Pain (BP) score was approximately 4 points lower during MCT intake than during LCT intake. Although it is possible that upper abdominal discomfort negatively influenced the subjective health status, no such discomfort was reported by participants. Thus, any impact was considered minimal.

## 5 Limitations

This study has several limitations. First, participants were aged 20–64 years. The effects in individuals younger than 20 years or 65 years and older remain unknown. Second, the trial enrolled individuals with a predisposition to constipation as defined by our eligibility criteria. Therefore, the effects of MCT intake in populations with normal bowel habits and in those who have fewer than two bowel movements per week or are diagnosed with chronic constipation according to the Rome IV criteria are undetermined. Third, for questionnaire outcomes, the SF-36 assesses the preceding month. As a result, the evaluation window included both the 2-week intervention and the 2-week pre-management period. Consequently, the results may be influenced, at least in part, by conditions during the pre-management period, which should be considered when interpreting the findings.

## 6 Conclusion

We conducted a randomized, double-blind, LCT-controlled, crossover trial to examine the effects of MCT intake on bowel function in Japanese adults aged 20–64 years with a tendency toward constipation, defined as three to five bowel movements per week. Using a statistical model to compare dietary effects within the same participants, significant diet effects were observed for three endpoints: number of days with bowel movements, bowel movement frequency, and stool volume. In addition, a *post-hoc* analysis confirmed that the change from baseline in the number of days with bowel movements at week 2 was significantly greater during the MCT intake period than during the LCT intake period. Collectively, these findings suggest that even a small amount of MCT (2 g/day) may contribute to improving bowel habits in constipation-prone Japanese adults. Moreover, given the low dose and the associated minimal participant burden, this intervention appears to be practical and potentially useful as a preventive dietary approach for constipation in everyday settings.

## Data Availability

The original contributions presented in the study are included in the article. Further inquiries can be directed to the corresponding author.
